# Parisian Medical Schools

**Published:** 1845-12

**Authors:** Alexander Wilcocks, R. M. Huston

**Affiliations:** 96 South Eleventh street; 96 South Eleventh street


### MEDICAL EXAMINER,

AND

### RECORD OF MEDICAL SCIENCE.

NEW SERIES—No. XII.—DECEMBER, 1845.

### ORIGINAL COMMUNICATIONS.

*96 South Eleventh street,*
*November 13th,* 1845.

Dear Sir,—You have asked me to give you some account of the facilities afforded at the present time for studying medicine in Paris; my best way of informing you upon this subject, is to give you a brief account of the twelve months which I have recently spent there myself.

The medical student in Paris begins the day at a hospital. Although by a rule of the "Bureau Central" the professors are required to commence their visits at six o'clock, A. M., in summer, and at seven o'clock, A. M., in winter, few of them are at their posts before eight o'clock.

Velpeau, who is the most popular surgeon in Paris, has generally a class of from twenty to fifty students, who follow him through the wards; he is from an hour to an hour and a half in making his visit. Velpeau says very little at the bedside; he there questions the patient, and examines his disease, but reserves all remarks for his lecture, which takes place immediately after the visit, in a small room adjoining his wards.

Owing to the number who follow this surgeon, it is impossible for many to receive much instruction during the *visit,* and this must be at the expense of a degree of pushing and scrambling which I have never seen practised at any hospital in this country.

Velpeau delivers his lecture with great distinctness, and for this reason American students are first taken to his wards by their countrymen. He begins by an account of those patients who have left the wards; he then speaks of the arrivals, and afterwards of any disease which may suggest itself, and finally of any operation or operations which he is about to perform. His operations always take place after the lecture. At ten o'clock he has finished his duties at the hospital. He gives a lecture at La Charité every day except Thursday.

Chomel, who stands in the first rank among the physicians, lectures three times a week at Hôtel Dieu, from November until April. During the remainder of the year Rostan has his chair. Both of these, but especially the latter, make many observations at the bedside, and their visits and lectures are exceedingly instructive; but the crowd is always a drawback. Besides these there are a number of other lecturers at various hospitals; Lisfranc and Berard, surgeons at la Pitié, and Piorry, a physician, at that hospital. Gibert and Devergie on diseases of the skin, at Saint Louis; Trousseau on the diseases of children, at Neckar; Civiale on calculous affections at the same hospital; and Ricord at Midi, &c. &c. All of these say much at the bedside, and a great deal of information may be obtained at their cliniques; but some of the lecturers waste their time, and that of their hearers, by abusing each other. Lisfranc, for example, never misses an occasion to express his antipathy for, and to ridicule Velpeau, for which the latter frequently retaliates.

When the morning lectures are out, (i. e. about 10½ o'clock) the students breakfast at one of the cafés in the neighbourhood of the hospitals; after this, those who are studying the rudiments of medicine go to the school; but this institution is very little frequented by Americans, who generally devote themselves to clinical studies. Between eleven and four o'clock, a number of private courses are given upon various medical subjects, some of them by agrégés of the faculty. These are held in different places, generally in the neighbourhood of the school. I will give you a list of those which I attended. Cazeaux on obstetrics, Sichel on the diseases of the eye, Desmanes on the same, Barth on pathological anatomy, Louget on the nervous system and on vivisections, and Ripbail on bandaging. But the great attraction which Paris formerly held out was the afternoon courses. Some of the internes at the hospitals were men of much experience, and of very industrious habits, and they enjoyed opportunities for studying the interesting cases which were withheld from persons not connected with the hospital. It was the custom for some of these internes to form a class of five or six foreigners, and pay a visit to the wards during the afternoon. An opportunity was thus afforded for any one to examine the cases at leisure, and practice himself in diagnosis, while the interne recapitulated any observations which the professor had made, and added any others with which his experience furnished him. But unfortunately all this is at an end. Last January, Orfila, the Dean of the Faculty, and the managers of the hospitals, put a stop to these private courses. The reason assigned for this was, that such repeated examination was injurious and cruel to the patients. But those internes who were engaged in the business said that the real opposition was on the part of their fellow internes, and arose from their jealousy.

While I was in Paris, I took one course on auscultation at Beaujon, in the service of Louis; two on surgery at La Charité, in the service of Velpeau; and two at this hospital, in Rayer's service. Besides these I had a course on the diseases of the skin at Saint Louis, in the service of Devergie; one at the Children's Hospital, in the service of Baudeloque; and one at St. Lazarre, on syphilis in women, and the exploration of the diseases of the neck of the uterus, by means of the speculum, and their treatment. St. Lazarre is not only a prison, but also an infirmary for those women who have venereal affections, but who are not on the register of the police.

These afternoon lectures ceased last January, much to the regret of all the foreigners, and if this privilege be not again granted, Paris has in my opinion lost its great charm for the American student.

I must recur again to the lectures in the middle of the day, to say that there are some subjects of which the professors make a speciality, which are not taught to any great extent in this country, viz.: The minute anatomy of the nervous system, and vivisections by Louget; pathological anatomy by Barth, and the diseases of the eye by Sichel, Desmanes, &c. &c. I must add the *touch* as applied to obstetric cases, and taught publicly by Dubois and Cazeaux. I omitted to say in the proper place that a large class followed Dubois in his visits to the wards of the "Clinique" of the school of medicine; and that the whole operation of labour and delivery may be witnessed by as many students as have tickets of admission to the clinique. These may be obtained by showing a diploma. The first four students who arrive in the delivery ward after labour begins, have the privilege of conducting the labour, in which no attempt is made to avoid exposure.

I have swelled this letter to a length which I never intended; but I cannot close without saying something about dissecting. All dissections in Paris are carried on in two places; the Ecole Pratique, which is close to the school, and Clamart, about two miles distant. The latter is probably the largest, cleanest, and best ventilated establishment of the kind in the world. The price of subjects varies with the season, but is generally from two to five francs. Lectures upon operations take place, and students may practice operating after the 1st of April; but this is forbidden before that time.

Whilst I was there I passed three months in the cabinet of the prosector to the faculty, M. Richet; I employed two of these in dissecting, and the other in operations. Any other information which I have upon these matters is freely yours.

With much esteem I remain, &c.,

ALEXANDER WILCOCKS.

PROF. R. M. HUSTON.

